# Editorial: Biomimetic scaffold materials for engineering of *in-vitro* tissue/organ models and *in-vivo* tissue repair

**DOI:** 10.3389/fbioe.2026.1985001

**Published:** 2026-09-21

**Authors:** Yi Wang, Zengxiao Cai, Lilith Caballero Aguilar, Gefei Chen, Linpeng Fan

**Affiliations:** 1 Chongqing Key Laboratory of Forage and Herbivore, College of Veterinary Medicine, Southwest University, Chongqing, China; 2 Institute for Frontier Materials, Deakin University, Geelong, VIC, Australia; 3 The Graeme Clark Institute, The University of Melbourne, Parkville, VIC, Australia; 4 Department of Biomedical Engineering, Faculty of Engineering and Information Technology, The University of Melbourne, Parkville, VIC, Australia; 5 Department of Medicine Huddinge, Karolinska Institutet, Huddinge, Sweden; 6 The Aikenhead Centre for Medical Discovery, Fitzroy, VIC, Australia

**Keywords:** biofabrication, biomimetic scaffolds, extracellular matrix, organoids, regenerative medicine, tissue engineering

## Introduction

Tissue defects caused by injuries and diseases continue to impair patients’ quality of life while placing an increasing burden on healthcare systems worldwide. To address these challenges, tissue engineering strategies employing biomimetic scaffolds have emerged as powerful approaches for repairing damaged tissues *in vivo* and engineering physiologically relevant tissue and organ models *in vitro*.

Recent advances in biomaterials have expanded the capabilities of biomimetic scaffold engineering. Emerging fabrication strategies have enabled more sophisticated control over scaffold structure and function. Improved understanding of cell-material interactions has promoted the development of biomaterials that not only support tissue formation but also actively regulate cellular behavior, immune responses and tissue regeneration.

The studies in this Research Topic reflect these advances across a broad range of biomaterials, fabrication technologies and biomedical applications. They demonstrate how innovations in scaffold engineering are improving the design of physiologically relevant *in vitro* tissue and organ models while promoting functional tissue regeneration *in vivo*.

In the following sections, we highlight several common emerging themes and discuss how the studies presented collectively advance the engineering and application of biomimetic scaffolds for both *in vitro* tissue modeling and *in vivo* tissue regeneration.

## Advances in biomimetic scaffold fabrication

Advances in scaffold fabrication continue to advance the development of biomimetic microenvironments. The studies presented in this Research Topic illustrate how complementary modern fabrication approaches, including self-assembly, electrospinning, additive manufacturing, and decellularization, are expanding the design possibilities of biomimetic scaffolds for tissue engineering and regenerative medicine.

Self-assembly remains one of the most versatile strategies for constructing biomimetic nanostructures under physiological conditions. As reviewed by Duc et al., self-assembling biomaterials can generate nanofibres, nanotubes and hydrogels that closely resemble the native extracellular matrix (ECM), while maintaining the structural simplicity and injectability that are desirable for minimally invasive therapies.

Electrospinning can be widely used for fabricating highly porous fibrous scaffolds that mimic the fibrillar architecture of native ECM. Beyond structural mimicry, Zhang et al. developed electrospun scaffolds incorporating biological functions, resulting in enhanced osteogenic differentiation.

Additive manufacturing has further broadened the capability of biomimetic scaffold engineering by enabling precise spatial control over scaffold geometry and composition. Three-dimensional (3D) printing and bioprinting provide versatile approaches for constructing patient-specific scaffolds while incorporating living cells and bioactive molecules, such as modular Ti-6Al-4V scaffold systems (Von Heckel et al.) and 3D printed composite scaffolds(Sanghvi et al.).

Nature-derived scaffolds continue to complement synthetic fabrication strategies by preserving many of the structural and biological characteristics of native tissues. For example, human acellularised amniotic membrane (HAAM) for corneal endothelial reconstruction by Chen et al.. Such biologically derived scaffolds provide valuable alternatives for engineering complex tissues in which preservation of ECM organization remains critical.

## Engineering cell-material interactions

Understanding how cells interact with biomaterials is fundamental to the successful design of biomimetic scaffolds. Beyond providing structural support, scaffolds actively influence cell adhesion, migration, differentiation, immune responses, and tissue organization through coordinated biochemical, topographical, and mechanical cues.

Cell adhesion represents the first step in establishing effective communication between biomaterials and surrounding tissues. Appropriate surface chemistry and microstructural organization determine not only initial cell attachment but also subsequent cellular activities. Ochima et al. showed that self-assembled monolayer-modified surfaces enhanced the adhesion and proliferation of cells. Similarly, modular Ti-6Al-4V scaffold systems showed that scaffold architecture and surface characteristics can direct fibroblast migration from the scaffold surface into the interior of 3D constructs (Von Heckel et al.).

Once cells establish contact with biomaterials, scaffold-derived biochemical and mechanical cues further influence cell fate and tissue development. Zhang et al. showed that sustained BMP2 gene delivery from electrospun scaffolds promoted osteogenic differentiation which is similar to porous silicon nanoparticles(Yin et al.), illustrating how scaffold-mediated delivery systems can regulate lineage commitment.

Another key development is the integration of immunomodulation into scaffold design. Spoddig et al. developed a 3D hydrogel co-culture system to reproduce key features of the foreign body response, providing a practical platform for evaluating implant materials while reducing reliance on animal models.

## Biomimetic scaffolds bridging *in vitro* tissue models and *in vivo* regeneration

One of the most notable themes is the close connection between biomimetic scaffolds developed for *in vitro* tissue engineering and those designed for *in vivo* tissue regeneration. Advances in scaffold design that improve the physiological relevance of engineered tissue models are simultaneously providing new strategies for regenerative medicine, while biological insights gained from regenerative studies continue to inform the development of more predictive *in vitro* models.

Biomimetic scaffolds play a central role in constructing physiologically relevant tissue and organ models by recreating the structural and biochemical complexity of native microenvironments. Duc et al. reviewed that engineered biomaterials advance neural models for neural development, disease modeling, and therapeutic screening. Engineered substrates also promote 3D culture and organoid formation (Ochima et al.).

These design principles are also guiding regenerative applications. Several studies demonstrate how biomimetic scaffolds regulate tissue repair by combining structural guidance with local biological signaling. HAAM further provides a biologically instructive scaffold for corneal endothelial reconstruction (Chen et al.), while stem cell-seeded biodegradable scaffolds consistently improve cartilage repair and tissue integration across preclinical models (Wróbel et al.). Collectively, these studies highlight the broad therapeutic potential of biomimetic scaffold technologies across diverse tissues and organ systems.

Advances in tissue models and regenerative applications are increasingly informing one another. Physiologically relevant tissue models provide powerful platforms for investigating disease mechanisms, evaluating biomaterial performance and optimizing scaffold design before animal studies and clinical translation. Regenerative applications continue to define the biological requirements that future tissue models must reproduce, including vascularization, immune regulation, tissue maturation and long-term functionality. This reciprocal interaction is accelerating the development of biomimetic scaffolds that are both biologically relevant and translationally meaningful.

